# Locomotion, Postures, and Substrate Use in Captive Southern Pygmy Slow Lorises (Strepsirrhini, Primates): Implications for Conservation

**DOI:** 10.3390/ani15111576

**Published:** 2025-05-28

**Authors:** Dionisios Youlatos, Dimitris Pylarinos, Nikolaos Evangelos Karantanis, Leszek Rychlik

**Affiliations:** 1 Department of Zoology, School of Biology, Aristotle University of Thessaloniki, 54124 Thessaloniki, Greece; dhm.pylarinos@gmail.com (D.P.); nekarantanis@gmail.com (N.E.K.); 2 International Center for Biodiversity and Primate Conservation, Dali University, Dali 671003, China; 3 Department of Ecology, School of Biology, Aristotle University of Thessaloniki, 54124 Thessaloniki, Greece; 4 Department of Systematic Zoology, Institute of Environmental Biology, Faculty of Biology, Adam Mickiewicz University, 61-614 Poznań, Poland; leszek.rychlik@amu.edu.pl

**Keywords:** canopy, forest, positional behavior, small branches, *Xanthonycticebus pygmaeus*

## Abstract

Studies of positional (=locomotor and postural) behavior are central to understanding how animals interact with the challenges imposed by their environment and are crucial for conservation management. The present study investigates, for the first time, the positional behavior and substrate use of the endangered southern pygmy slow loris *Xanthonycticebus pygmaeus*. Despite their very specialized morphology and ecology, the positional behavior of lorises is understudied. Behavioral data were collected using 30-s scan instant sampling on seven captive animals housed in a large, enriched enclosure of the Poznań Nowe Zoo (Poland) during February–June 2013. Pygmy slow lorises were almost exclusively arboreal and most activities occurred on multiple substrates (82.93%). Small (57.91%) and large (28.28%) substrates were extensively used. Horizontal (42.11%) and oblique (38.47%) substrates dominated. Clamber (39.39%), quadrupedalism (33.77%), and vertical climb (17.62%) were the main locomotor modes. Standing was the dominant posture (47.23%), followed by hanging (27.32%) and clinging (11.31%). Our results concur with the limited observations available on other lorisines; pygmy slow lorises employed a diverse and flexible positional repertoire as an adaptation to the exploitation of the continuous forest layers with intertwined small substrates of various inclinations. Consequently, protecting and managing these habitats, supported by ex situ reintroduction programs, can effectively contribute to the conservation of the species’ populations.

## Simple Summary

This study presents the first detailed investigation of the positional behavior and substrate use of the endangered southern pygmy slow loris (*Xanthonycticebus pygmaeus*). Behavioral data were collected from seven captive individuals housed at the Poznań Nowe Zoo (Poland) between February and June 2013. The results revealed that pygmy slow lorises are almost exclusively arboreal, particularly favoring small, horizontal, and oblique substrates. Major locomotor behaviors included clamber, quadrupedalism, and vertical climb, while the primary postures observed were standing, hanging, and clinging. These findings align with limited previous observations on other lorises and emphasize the species’ highly adaptable and diverse locomotor repertoire, enabling them to navigate complex forest structures with intertwined, variably inclined substrates. The study underlines the importance of conserving such habitats and enhancing by ex situ reintroduction programs to support the effective conservation of pygmy slow loris populations across their natural range.

## 1. Introduction

Locomotion, or active body displacement, and postures, or dynamic stability of the body, enable animals to interact effectively with their surrounding environment. The different modes of displacement and stance represent the locomotor and postural behavior, respectively, and are collectively referred to as positional behavior [1]. Positional behavior represents a crucial biological factor, as it provides access to and handling of food sources, helps escape from predators, enables dominance and territorial displays, and facilitates active searching and interaction with potential mates, contributing to reproduction and survival [2]. Thus, studies of positional behavior are central to animal biology, elucidating how animals behaviorally adapt to their environments. These eco-mechanical variables present biological information that is important for advances in evolutionary and adaptive morphology, fossil reconstruction, life history traits, and ecology [2]. From an evolutionary-adaptive perspective, studies of positional behavior are integral to interpreting how organismal and ecological traits have evolved to help animals increase their fitness, thereby interacting within the constraints of their environment. In addition to traditional evolutionary analyses, considering the relationship between positional behaviors and effective habitat use in conservation terms is critical [3,4]. It informs how animals respond to varying environmental conditions to survive and reproduce [5]. Consequently, this information can be vital for conservation planning, habitat management, and captive breeding or reintroduction programs [3,4,5,6].

In this context, studies of positional behavior are particularly important when considering endangered and less-studied animals, such as the lorises (Lorisidae, Strepsirrhini, Primates) of South and Southeast Asia. Lorises include three genera of nocturnal strepsirrhine primates: *Loris*, the slender lorises with two species; *Nycticebus*, the slow lorises comprising eight species; and the slow pygmy lorises *Xanthonycticebus*, recently recognized as a separate genus containing two species [7,8]. Except for the gray slender loris (*Loris lydekkerianus*), which is listed as Near Threatened (NT), all other loris species are categorized as threatened by the IUCN: three species are classified as Vulnerable (VU), five as Endangered (EN), and one as Critically Endangered (CR). This study investigates the positional behavior of captive pygmy slow lorises, currently classified as Endangered (EN) by the IUCN [9]. The primary threats of this species include habitat degradation and loss, illegal hunting, pet trade, and use in traditional medicine [9,10,11]. The southern pygmy slow loris, *Xanthonycticebus pygmaeus* (Bonhote 1907), is one of the two species of the recently recognized genus *Xanthonycticebus* and most likely diverged early (between 6 and 26 Ma) from the rest of slow lorises [7,8]. The species inhabits the eastern part of Indochina, ranging from South China to Vietnam, Laos, and Kampuchea. It has a mean body mass of approximately 424 g, a round face with an intermediate interorbital distance, a reduced index digit, a very large third hand pad, full coat seasonal change, and lacks significant sexual size dimorphism [7,9,10,12]. Southern pygmy slow lorises inhabit a wide variety of habitats, including primary evergreen and semi-evergreen forests, limestone forests, secondary and highly degraded habitats, and bamboo thickets [9,10]. They are nocturnal obligate exudativores, with over 50% of their diet composed of gums extracted from tree barks via their derived toothcomb morphology; their diet is further supplemented by arthropods, fruit, and other plant or animal matter [10,13,14]. The species forages alone or in small groups of four individuals, uses primarily solitary sleeping patterns except for females who sleep with their young, and displays a multi-male, multi-female social system [9,14,15,16,17]. They are seasonal breeders, giving birth to twins in the winter months [9,10,16]. They are among the few venomous mammals, which is most likely related to ectoparasite control, antipredator deterrence, and intraspecific competition [18,19].

Asian lorises exhibit distinctive morphological traits, such as few caudal vertebrae, a relatively high number of thoracic vertebrae, expanded ribs, large and mobile joints in the humerus and femur, very flexible wrist and ankle joints, strong grasping hands and feet with a wide first digit abduction and a reduced second manual digit, as well as frontated and upwardly rotated orbits with relatively high orbital convergence [20,21,22,23,24,25,26,27,28,29,30]. They move in a slow, fluid, and acrobatic way, do not leap, and use freezing postures when disturbed [26,29,31,32,33,34,35,36,37,38,39,40,41]. However, the relationship between their locomotor and postural behaviors and habitat use has not been thoroughly investigated, primarily due to their cryptic and nocturnal nature.

Only a limited number of studies have examined the positional behavior of a small number of loris species, both in the wild and in captivity. The slender lorises *Loris lydekkerianus* and *L. tardigradus* have been already studied in the wild [35,36,39]: Both species commonly use quadrupedalism (50–58%), with higher rates of faster bouts in the latter (26% vs. 5%), along with lower percentages of climbing activities (30–40%) and considerable rates of bridging and suspensory modes (6–19%). In terms of postural behavior, both species use the sitting posture at very high rates (70–76%), with moderate rates of standing (8–16%), clinging (~10%), and hanging postures (5–12%). Finally, both species habitually use small (44–75%) and inclined substrates (42–73%). *Loris tardigradus* makes substantial use of vertical substrates (19–22%), seldom used by *L. lydekkerianus* (~3%) [35,36,39]. In the wild, the slow lorises *Nycticebus javanicus* and *N. bengalensis* emphasize climbing activities (55% and 56%, respectively), use quadrupedalism to a lesser extent (18% and 36%, respectively), but mainly at slower bouts, and bridging/hanging at even lower percentages (17% and 8%, respectively) [40,41]. Within postural modes, sitting and ball postures are commonly employed (55% and 76%, respectively), followed by standing (18% and 10%, respectively), clinging (9% for *N. javanicus*), cantilevering (~3% and 8%, respectively), and hanging (18% and 4%, respectively). In terms of substrate use, *N. bengalensis* mainly utilize medium (62%) and inclined (75%) substrates, with moderate use of either horizontal (14%) or vertical substrates (10%) [41]. Finally, *N. coucang*, studied in captivity [31,32], divides its locomotor behaviors between quadrupedalism (43%) and climbing (49%), while bridging/hanging are rather uncommon (~8%). Standing/crouching is the most common posture (67%), along with sitting (22%), and hanging (9%) [32]. Moreover, the use of horizontal substrates dominated (50%), along with a moderate use of vertical ones (14%) [31].

To our knowledge, there are no studies conducted on the locomotion and postures of the southern pygmy slow loris (*X. pygmaeus*) in the wild or in captivity (but see [42]). To address this gap, we conducted a quantitative study on the species’ locomotion, postures, and substrate use in an enriched enclosure at the Nocturnal Pavillon of the Poznań Nowe Zoo (Poland). Based on existing information on the species, which indicates a predominant reliance on an arboreal environment, characterized by fluid, continuous, acrobatic locomotion and postures primarily on small and on vertical substrates [7,9]. We expect to find (i) a high degree of arboreality; (ii) increased rates of climbing/clambering activities, reduced quadrupedalism, and lack of leaps; (iii) frequent use of standing/crouching, clinging, and hanging postures; (iv) common use of vertical substrates, particularly during feeding activities; (v) habitual use of small substrates, especially during moving and feeding behaviors. Such information will offer insights into the anatomical specializations of the species and highlight the microhabitat features that need to be preserved for effective conservation and management.

## 2. Materials and Methods

We conducted our research in full compliance with the IUCN Commission Statement on Research Involving Species at Risk of Extinction [43], the Code of Best Practice in Field Primatology and the Principles for the Ethical Treatment of Primates, established by the American Society of Primatologists [44], and the Guidelines for the Treatment of Animals in Behavioral Research and Teaching [45]. Additionally, our study met the regulations of the Poznań Nowe Zoo and the Adam Mickiewicz University in Poznań, as well as the ethics legislation of the Aristotle University of Thessaloniki.

### 2.1. Study Animals and Experimental Setting

**Figure 1:**
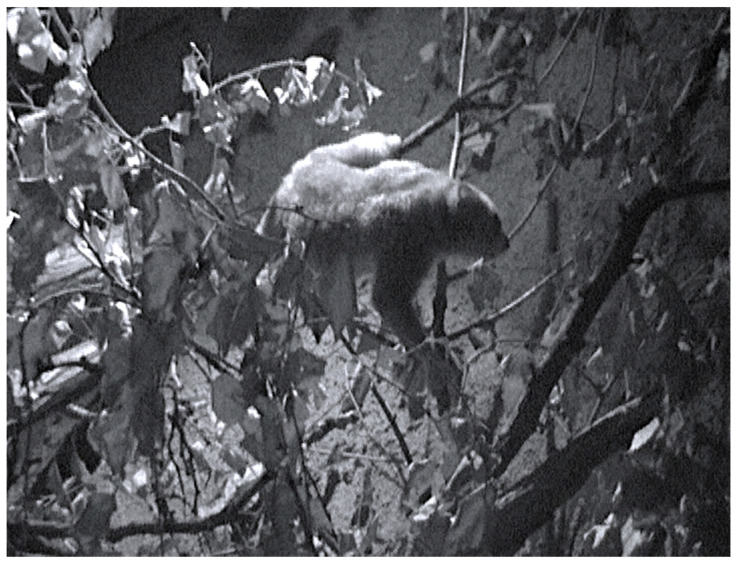
Video still of a study animal clambering around the surrounding environment of the enclosure.

For the purposes of the current study, we observed and filmed three male and four female captive, adult southern pygmy slow lorises, *Xanthonycticebus pygmaeus*, in the Nocturnal Pavillon of the Poznań Nowe Zoo (Poznań, Poland). All study animals were captive-born, healthy, fully habituated to human presence, and did not display any stereotypical behaviors during the study period. At the time of observations, the animals were housed together in an enclosure (H: 280 cm × W: 340 cm × D: 240 cm) in the display colonies of the Nocturnal Pavilion of the zoo under a reversed day–night regime. The front of the enclosure was covered by a large glass window, whereas the sides were covered by concrete walls, and the floor was covered with dirt. The enclosure contained a wide variety of intertwined available substrates of diverse sizes (thin twigs to wide chunks of trunks) and inclinations (horizontal to vertical), enabling the animals to move freely in a three-dimensional, enriched environment [46] (see Figure 1). Available food consisted of mealworms, crickets, and peeled and cubed fruit placed on wooden ledges and in hanging feeders, enabling regular ad libitum feeding of the study animals.

Despite the enriched arboreal environment, substrates in an artificial enclosure are always expected to limit the locomotor and postural options of caged animals. In this case, estimating substrate availability allows for a controlled, weighted test of substrate preference. We calculated all available substrates by unit and, subsequently, estimated the availability of the different size and inclination categories (see Table 1 for definitions). Small substrates dominated (44.6%), with medium and large ones ranking behind (31.4% and 24.0%, respectively). Regarding substrate inclination, oblique substrates were more abundant (53.1%), followed by horizontal (26.3%) and vertical ones (20.6%). Preference or avoidance of these categories was then estimated utilizing Jacobs’ index: D = U − A/U + A − 2U × A, where U is proportion of use and A is proportion of availability. Values of the index range from −1, depicting strong avoidance, to +1, showing strong preference, whereas values around 0 are considered as neutral [47].

A relatively rich enclosure housing a significant number of animals of both sexes has been found to be beneficial for promoting a diverse repertoire of behaviors and associated locomotor and postural modes [46]. According to these authors, multi-sex groupings in moderately sized enclosures may enhance social proximity and interactions as well as grooming frequency while also reducing moving, all without significantly influencing positional behaviors [42]. Therefore, we consider this enclosure to provide a suitable context for exploring the diversity of locomotor and postural modes and their relationship to habitat features in southern pygmy slow lorises.

### 2.2. Data Collection

The present data derive from the analysis of extensive video recordings (February to June 2013) of the study animals on days when the zoo was closed to the public. This allowed direct and uninterrupted access to the animal enclosure without external disturbance. Consequently, the animals were filmed twice per week from 10:00 to 17:00, using additional infrared lighting. During video recording sessions, we used a SONY Hi-8 CCD-TR705E (Sony Corporation, Tolyo, Japan) camcorder at 24 fps and at a shutter speed of 1/500th. The original Hi-8 tapes, totaling 25 h of recordings, were digitized and subsequently analyzed frame-by-frame on a PC for data collection.

During video analyses, we used the 30 s scan instant sampling method for recording behavioral observations from all different visible animals [48]. For slow-moving animals, such as the pygmy slow loris, 30 s instants allow for independence of succeeding behavioral events. During each 30 s instant, we recorded the following variables: (i) substrate type, (ii) substrate size, (iii) substrate inclination, (iv) substrate number, (v) behavioral context, and (vi) locomotor or postural mode (see Table 1 for the different categories and their definition). Finally, although positional modes and substrate use usually relate to behavioral contexts (e.g., feed, travel, etc.) in the wild, a captive setting, with its spatial limitations and specific feeding conditions, usually modifies and introduces biases with similar associations, and therefore were not included in the current study.

### 2.3. Data Analysis

During the data collection process, we paid particular attention to equally sampling all seven individuals, resulting in a mean of 3364 ± 128 instantaneous samples (range: 3142–3469 instantaneous samples). For each sampled individual, the data were arranged in tables and the frequencies of the different categories were calculated. Inter-individual variability was tested with the Wilk’s lambda (*λ*) test [49]. We found no significant differences between individual profiles for all the tested variables (Wilk’s *λ* = 0.128, *p* = 0.881). For this reason, we combined all data from the seven animals and created a large dataset (pooled total = 23,551). We then calculated percentages for each variable and compared differences in percentages of use with the non-parametric log-likelihood ratio G test, as similar data are not arranged in a natural way, violating assumptions of randomness and departing from normality [50]. All statistical analyses were performed in SPSS 25.0 (IBM SPSS Inc, New York, NY, USA). *p*-values ≤ 0.05 were regarded as statistically significant and only those are reported in the Section 3.

## 3. Results

### 3.1. Substrate Use

During the study period, the lorises were almost entirely arboreal (99.53%), descending on the enclosure floor only very rarely. During arboreal activities, branches were primarily used (71.87%).

**Figure 2:**
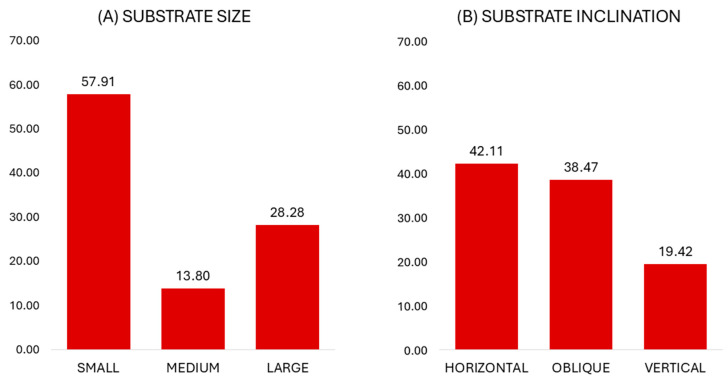
Percentages of substrate size and inclination use in captive *Xanthonycticebus pygmaeus*.

When on arboreal substrates, the animals primarily used and preferred small branches (57.91%, Jacob’s D = 0.15), whereas large substrates were also considerably used (28.28%) and preferred (Jacob’s D = 0.26) (Figure 2).

In terms of substrate inclination, the captive lorises frequently used and preferred horizontal substrates (42.11%, Jacob’s D = 0.42) (Figure 2). The use of vertical substrates was also considerable and according to availability (19.42%, Jacob’s D = 0.06).

Throughout the study, the lorises mainly used multiple substrates (82.93%), while single substrates were only occasionally used (17.07%).

### 3.2. General Behavior

During the study period, the lorises largely divided their behavior between resting (33.23%) and moving (31.21%). Grooming behavior was considerably present (17.83%), whereas feeding activities represented 11.96% of all behaviors. Social interactions were not very common (5.76%).

When moving, the study animals primarily used small and oblique substrates (Table 2). Substrate use during feeding was similar, with slightly increased use of vertical substrates (Table 2). In contrast, while resting, large and horizontal-dominated (move vs. rest, substrate size use: G = 47.5, *p* < 0.001; substrate inclination use: G = 44.2, *p* < 0.001; feed vs. rest, substrate size use: G = 23.6, *p* < 0.001; substrate inclination use: G = 31.7, *p* < 0.001). During grooming and social activities, the lorises similarly remained on large and horizontal substrates (Table 2).

### 3.3. Locomotor Behavior

**Figure 3:**
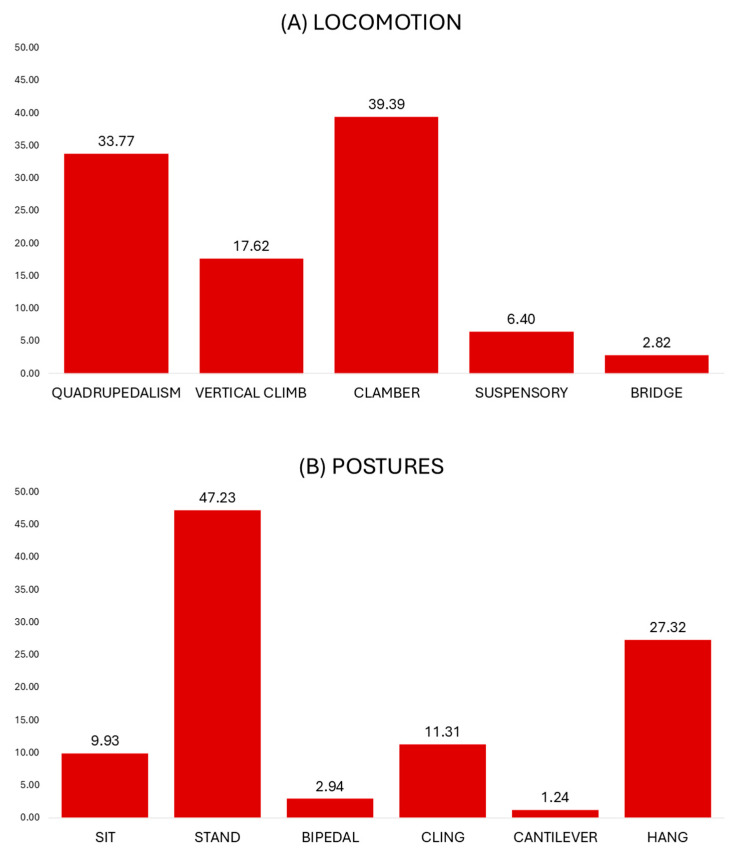
Percentages of locomotor and postural modes used in captive *Xanthonycticebus pygmaeus*.

The locomotor profile of the lorises is shown in Figure 3A. The main locomotor mode was clambering (39.39%). The animals moved cautiously across multiple branches of diverse inclinations, maneuvering their limbs at variable angles, rotating their bodies, and strongly grasping the available substrates. Clambering occurred mainly on small and medium substrates (Table 3). This profile is significantly different from almost all other locomotor modes, except in inclination use with bridge (clambering vs. quadrupedalism, substrate size use: G = 321.4, *p* < 0.001; substrate inclination use: G = 428.6, *p* < 0.001; clambering vs. vertical climbing, substrate size use: G = 181.4, *p* < 0.001; substrate inclination use: G = 349.5, *p* < 0.001; clambering vs. suspensory locomotion, substrate size use: G = 46.1, *p* < 0.001; substrate inclination use: G = 92.9, *p* < 0.001; clambering vs. bridging, substrate size use: G = 16.7, *p* < 0.001).

Quadrupedalism was the second-most frequent locomotor mode (33.77%) and involved regular swinging and stance phases of the limbs upon single horizontal and moderately inclined substrates. Most of these bouts were slow (92.87% of quadrupedal bouts), while rapid locomotion was only occasional (7.13% of all quadrupedal bouts). Quadrupedal bouts were most common on large and horizontal substrates (Table 3; quadrupedalism vs. vertical climbing, substrate size use: G = 21.8, *p* < 0.001; substrate inclination use: G = 736.2, *p* < 0.001; quadrupedalism vs. suspensory locomotion, substrate size use: G = 43.5, *p* < 0.001; substrate inclination use: G = 8.4, *p* = 0.003; quadrupedalism vs. bridging, substrate size use: G = 11.7, *p* = 0.003; substrate inclination use: G = 86.5, *p* < 0.001).

Vertical climbing, involving regular swinging and stance phases of the limbs upon vertical and steeply inclined substrates, was considerably used (17.62%). Vertical ascent and vertical descent were almost equally used (50.39% and 49.61% of all vertical climbing bouts). Vertical substrates and large and medium substrates were commonly used (Table 3; vertical climbing vs. suspensory locomotion, substrate size use: G = 13.1, *p* = 0.001; substrate inclination use: G = 303.8, *p* < 0.001; vertical climbing vs. bridging, substrate size use: G = 6.4, *p* = 0.04; substrate inclination use: G = 82.4, *p* < 0.001).

Suspensory locomotion below branches was moderately used (6.40%). During suspensory activities, most bouts involved supinograde (inverted) quadrupedal locomotion along branches (91.46% of all suspensory locomotion bouts). Suspensory locomotion occurred predominantly on medium and horizontal substrates. Bridging activities took place on similar substrates in terms of size, but on inclined rather than horizontal substrates (Table 3; suspensory locomotion vs. bridging, substrate inclination use: G = 39.3, *p* < 0.001). Bridging gaps by extending and reaching the limbs and the body towards terminal substrates was rather infrequent (2.82%). During bridging, small and oblique substrates were mostly used. No leaping activities were observed during the study.

### 3.4. Postural Behavior

The postural profile of captive lorises is shown in Figure 3B. During the study period, the most common posture was standing (47.23%). Most standing bouts were represented by pronograde quadrupedal standing with partly flexed limbs (66.77% of all standing bouts), whereas the crouched posture, involving strongly flexed limbs, was also substantially used (33.23% of all standing postures). Standing mainly occurred on large and on horizontal substrates showing significant differences with substrate size and inclination use of other postures (Table 4; standing vs. sitting, substrate size use: G = 60.7, *p* < 0.001; substrate inclination use: G = 48.3, *p* < 0.001; standing vs. hanging, substrate size use: G = 251.6, *p* < 0.001; substrate inclination use: G = 214.8, *p* < 0.001; standing vs. clinging, substrate size use: G = 113.6, *p* < 0.001; substrate inclination use: G = 920.8, *p* < 0.001; standing vs. bipedal, substrate size use: G = 87.9, *p* < 0.001; substrate inclination use: G = 60.1, *p* < 0.001; standing vs. cantilevering, substrate size use: G = 37.6, *p* < 0.001; substrate inclination use: G = 152.4, *p* < 0.001).

Hanging was the second-most frequent posture in our study (27.32%, Figure 3B). During hanging postures, the body was partly or fully suspended in a flexible supinograde or semi-pronograde position from any combination of fore- and hindlimbs. Hanging by all fours was the dominant position (72.13% of all hanging postures). Hanging postures occurred predominantly below small and oblique substrates (Table 4; hanging vs. sitting, substrate size use: G = 55.4, *p* < 0.001; substrate inclination use: G = 68.6, *p* < 0.001; hanging vs. bipedal, substrate size use: G = 37.1, *p* < 0.001; substrate inclination use: G = 45.4, *p* < 0.001; hanging vs. cantilever, substrate size use: G = 9.1, *p* = 0.01; substrate inclination use: G = 56.1, *p* < 0.001).

The clinging posture was used considerably (11.31%, Figure 3B). During clinging, on most occasions, the body was positioned with the head up close to the strongly inclined substrate (58.64% of all clinging postures). The use of small and vertical substrates dominated (Table 4; clinging vs. sitting, substrate size use: G = 29.7, *p* < 0.001; substrate inclination use: G = 554.7, *p* < 0.001; clinging vs. bipedal, substrate size use: G = 28.3, *p* < 0.001; substrate inclination use: G = 245.5, *p* < 0.001; clinging vs. hanging, substrate inclination use: G = 408.6, *p* < 0.001; clinging vs. cantilevering, substrate size use: G = 6.2, *p* = 0.04; substrate inclination use: G = 9.1, *p* = 0.002).

The sitting posture was occasionally used and occurred primarily on large and oblique substrates (Table 4). Standing, however, took place mostly on large and horizontal substrates (sitting vs. bipedal, substrate size use: G = 19.7, *p* < 0.001; substrate inclination use: G = 12.5, *p* < 0.001; sitting vs. cantilevering, substrate size use: G = 23.7, *p* < 0.001; substrate inclination use: G = 115.8, *p* < 0.001).

The bipedal posture (2.94%) and cantilevering were rarely used (1.24%) and differed significantly in substrate size and inclination use (Table 4; bipedal vs. cantilevering, substrate size use: G = 15.7, *p* < 0.001; substrate inclination use: G = 67.8, *p* < 0.001).

## 4. Discussion

To our knowledge, this is the first study of the positional behavior of southern pygmy slow lorises *Xanthonycticebus pygmaeus*. A previous study in captivity by [42] included combinations of positional modes that are difficult to compare with this study. Our study was conducted in a captive setting, which is expected to limit the options of locomotor and postural modes and substrate use. However, the size of the enclosure, the enhanced variety of the available arboreal substrates, the multi-sex grouping of the animals, as well as the ad libitum feeding of the animals assured a diversity of activities that may partly approximate the natural behaviors of the species [42,46]. In general, the pygmy slow lorises were almost exclusively arboreal. Moreover, they primarily moved through clambering and quadrupedalism and employed standing and hanging postures. In terms of substrates, they extensively used small, horizontal, and oblique substrates. Small and horizontal were strongly preferred. Small substrates were primarily used during feeding activities and movement. Oblique substrates were also used during both behaviors, while vertical substrates showed relatively increased rates only during feeding. These findings appear to support most of the predictions outlined in this study.

The prevalence of arboreal behaviors of *X. pygmaeus* in this study reinforce previous observations indicating that pygmy slow lorises are mostly encountered in the canopy [10,51,52,53]. Pygmy slow lorises are present in a variety of habitats, including primary evergreen, semi-evergreen, secondary forests, or bamboo thickets [9], where they exploit the dense canopy of intertwined branches. As such, pygmy slow lorises are similar to other studied lorisines, being almost exclusively arboreal and descending rarely on the ground to cross gaps in very discontinuous habitats [29,41] and to travel across forest patches at moderate speeds [51]. Rare excursions on the enclosure floor were also observed in our study during traveling and exploring the enclosure.

The locomotor profile of *X. pygmaeus* supported our prediction and is comparable to that of other lorisines. Previous studies on captive slow lorises *Nycticebus coucang* show shared rates between quadrupedal and climbing (which includes our clamber category) activities [32,54]. Glassman and Wells [32] also reported some anti-pronograde behaviors (e.g., pull-up) most likely related to our categories of bridging and suspensory locomotion. However, as with the case of captive *X. pygmaeus*, they were not very common. More recent and detailed studies of wild *Nycticebus javanicus* [40] and wild *Nycticebus bengalensis* [41] described comparable locomotor profiles for these species. Clambering/climbing activities seem to dominate over quadrupedalism, representing over half of all locomotor modes, especially in *N. javanicus* (64.8%, [40]). The latter species also used increased rates of bridging (11.2%) and suspensory locomotion (5.7%), whereas these behaviors were more restricted in *N. bengalensis* (8.0%, [41]). Increased rates of bridging and suspensory behaviors in these species may be related to the effective exploitation of their natural habitats. These studies were conducted in patches of cultivated land, bamboo, and shrubs in Cipaganti, Indonesia [40], and semi-evergreen forests, interspersed with wet evergreen forest patches with several canopy layers, in the Hollongapar Gibbon Wildlife Sanctuary, India [41]; both locations are composed of a discontinuous canopy that compels animals to slowly cross between gaps when traveling. As lorisines do not leap (an observation further substantiated in our study), bridging represents the main means to cross canopy gaps usually abundant in forest canopies. However, in a captive setting, gaps are usually smaller in size, and animals prefer to opt for safer ways to cross them, such as clambering, climbing, or quadrupedalism, depending on the availability and architecture of the initial and terminal substrates.

The relation of bridging behavior to a natural canopy setting is further substantiated by the relatively high rates in the wild slender lorises *L. lydekkerianus* (13.6%: [35]; 19.1%: [36]) and *L. tardigradus* (6.2%: [36]; 7.0%: [39]). Moreover, slender lorises seem to differentiate from both slow lorises and the pygmy slow loris in two ways. (a) Slender lorises show higher percentages of quadrupedal activities, ranging between 58% in *L. tardigradus* [39] and 50.2% in *L. lydekkerianus* [35]. These rates are considerably lower in wild *Nycticebus* spp. [40,41] and in *X. pygmaeus* (this study). The increased rates of quadrupedalism recorded in earlier captive studies [32,54] may be related to either the design of the captive setting, arranged in a less enriched way, or to mode definition differences. Further studies using common definitions of positional modes are required to identify such discrepancies.

Additionally, some slender lorises are faster than other lorises. In *L. tardigradus*, rapid quadrupedalism represents a considerable proportion of the quadrupedal sample (37.2%: [36]; 44.8%: [39]). However, rapid quadrupedalism is uncommon in *L. lydekkerianus*, representing only 8.7% of all quadrupedal bouts [36], similar to that for *N. javanicus* (5.5%, [40]) and *X. pygmaeus* (7.1%, this study). It is very likely that the short and particularly muscular limbs, the powerful grasping abilities, and the flexible ankle and wrist joints may hinder the development of higher speeds and the common use of rapid locomotion in slow lorises [12,20,21,22,23,24,25,26,27,28,29,30,38]. Comparative functional studies between *Nycticebus*, *Xantonycticebus*, and *Loris* will certainly shed light to this end. Furthermore, in the case of pygmy slow lorises, the captive setting may have further prevented the animals from achieving higher speeds. More studies in the wild are required to better understand any differences in speed of locomotion between slender, slow, and pygmy slow lorises.

Regarding postural behavior, standing was the main posture of *X. pygmaeus* and included quadrupedal stances with both semi-extended and very flexed (crouch) limbs. This finding supported our initial prediction. Comparable standing percentages (67%) were observed in captive *N. coucang* [32]. In contrast, all other studies of lorisines demonstrated much lower proportions of standing. Tenaza et al. [54] found that standing/crouching represented only 12.1% of postural behavior of captive *N. coucang*, whereas similar results were obtained for wild *N. javanicus* (18.5%) [40] and wild *N. bengalensis* (10%) [41]. Equally low percentages have been also recorded for wild *L. tardigradus* (16.7% [36]) and wild *L. lydekkerianus* (9.9% [35]; 8.1%, [36]). Standing postures are usually related to more active behaviors, such as exploring, feeding, and pausing between locomotor bouts. In contrast, almost all studies on lorisines emphasize the use of sitting, representing up to 70% of all postural behaviors [35,36,40,41,54]. This posture, especially in the form of a ball with the body hunched and the head erect between the knees, is related to longer and more stable bouts of inactivity, adopted during either resting, sleeping, or grooming behaviors. It is very likely that our sampling protocol underestimated it during our captive study. Since wild lorisines use this posture at sleeping sites for thermoregulation, during colder nights ([29]; see also [55]), the stable environmental conditions in captivity likely reduced the need for comparable behavioral adaptations.

As predicted, pygmy slow lorises frequently used hanging postures. This is similar to previous observations, albeit much more amplified even when the transitional posture of other studies is also included, which are as follows: captive *N. coucang* (9.8%, [32]), wild *N. javanicus* (14.1%, [40]), wild *L. lydekkerianus* (15.7%, [36]), and wild *L. tardigradus* (12.5%, [36]). Hanging postures, in combination with the small body mass of pygmy slow lorises, enable the efficient use of small, inclined, and flexible substrates [41]. Safely anchored by the increased grasping ability of the muscular extremities and agilely positioned by the enhanced mobility of the fore- and hindlimb joints [20,21,22,23,24,25,28,30], these postures facilitate the exploration of tree peripheries, increasing access to potential food sources, such as insects and small fruit, and ensures the initiation of safe crossings between small gaps in the canopy.

Moreover, as hypothesized, pygmy slow lorises considerably used clinging postures. In this context, our findings resemble previous observations of other lorisine species, such as wild *N. javanicus* (9.3%, [40]) and wild *L. lydekkerianus* (9.9%, [35]. In our study, this type of posture is related to the use of large and vertical substrates. Within their natural habitat, pygmy slow lorises likely adopt clinging postures to feed on tree gum, which constitutes a significant portion of their diet [10,13,14,56]. Although no gum feeding occurred in the captive setting, clinging postures enabled the animals to hold on to large vertical substrates and collect food from the vertically hanging feeders. The frequent use of vertical substrates, especially during feeding behavior, aligns with our initial predictions. Overall, vertical substrate use in *X. pygmaeus* was comparable to that of *L. tardigradus* (19.5%: [36]; 22% [39]) but higher than in *L. lydekkerianus* (2.9%, [36], *N. bengalensis* (10.6%, [41], and *N. coucang* (14%, [31]). Interestingly, during feeding activities, vertical substrate use significantly increased to 30.09%, likely linked to gum feeding [10,52,56]. The strong muscular limbs and the efficient prehensile extremities of pygmy slow lorises [20,22,28] enable grasping on vertical substrates, maintaining a prolonged clinging posture.

Southern pygmy slow lorises extensively used and preferred small substrates. As hypothesized, their usage rates remained high during moving and feeding activities compared to other behaviors, where larger substrate usage prevailed (Table 2). Comparison with previous studies is difficult due to the different substrate size definitions. Slow lorises (*N. bengalensis*, *N. javanicus*) mainly use medium branches (56%, [57]; 62.3%, [41]; Nekaris, unpublished data). In contrast, most studies on slender lorises support our findings: wild *L. lydekkerianus* (69.5%, [36]), wild *L. tardigradus* (75%, [36]; 44%, [39]). In general, substrate size utilization is typically constrained by body mass, with larger primates selecting larger branches, while smaller primates can exploit a broader range of available sizes. Like small-bodied slender lorises (*L. tardigradus*: 105–170 g; *L. lydekkerianus*: 225–320 g; [29]), the relatively small body mass of pygmy slow lorises (360–580 g) allows them to move and feed securely and efficiently on fine branches. Their ability to effectively utilize small branches through clambering, bridging locomotion, and hanging postures enables them to navigate dense forest canopies safely, cover relatively long daily ranges, and access diverse food sources [9,10,13,14].

The findings of our study provide insights into the ecological niche of southern pygmy slow lorises, which is crucial for designing and implementing conservation actions [3,4,5,6,41]. Our study shows that southern pygmy slow lorises are specialized arboreal dwellers emphasizing clambering, quadrupedalism, and standing and hanging postures associated with fine-scale canopy architecture. As habitat degradation and loss are major threats to the species, effective long-term, in situ conservation must extend beyond simply protecting forested areas. It should also focus on restoring and preserving the structural and compositional complexity of these habitats. In this way, priority should be given to the preservation of habitats with a high density of small, horizontal, and oblique branches, which *X. pygmaeus* frequently use for both locomotion and feeding. More particularly, small branches are critical, allowing southern pygmy slow lorises to use their clambering locomotion and hanging postures, enabling successful navigation and effective access to variable food sources within the canopy [9,58]. These substrates should be preserved or restored in both the primary evergreen and semi-evergreen forests, as well as the regenerating secondary forests, ensuring that habitat quality is suitable.

Moreover, it is also important to maintain or restore the vertical complexity of the forest, especially the presence of large vertical trunks and/or lianas, as these encourage clinging postures commonly used during feeding, particularly gum extraction, a vital dietary component [10,13,14,56]. The continuity of the canopy is also of primary significance, as the southern pygmy slow loris relies on bridging locomotion to cross gaps. In primary forests, a multilayered canopy with intertwined crowns should ensure this three-dimensional network of continuousness. However, in degraded and fragmented habitats, the creation of arboreal bridges, vegetated corridors, or canopy highways can significantly enhance connectivity between forest patches, allowing for safe dispersal and access to diverse feeding sites [59]. The preservation of these specific architectural features of forested environments will most likely aid this endangered species in efficiently exploiting their habitat assuring their survival and reproduction.

On the other hand, illegal capture for pet trade, traditional medicine, or subsistence often leads to significant physical and psychological harm, rendering individuals unfit for immediate release into the wild. Recovery requires the controlled and safe environment of captive breeding, rehabilitation and reintroduction centers. In captivity, enclosures should offer a complex network of small, horizontal, and oblique arboreal substrates, where the animals can engage in clambering, quadrupedal activities, as well as standing and hanging postures [31,42,46]. Such enrichment enhances behavioral recovery and fitness [41,42], ultimately improving survival prospects upon reintroduction in preserved areas.

## 5. Conclusions

This is the first detailed study of the positional behavior of the endangered southern pygmy slow loris *X. pygmaeus*. Although this study was conducted in a captive setting, it provides valuable information on how this species interacts with specific habitat features. Our results demonstrate the arboreal nature of the species, with clambering and quadrupedalism as the main locomotor modes, and standing and hanging as the dominant postures. Moreover, captive southern pygmy slow lorises extensively used small, horizontal, and oblique substrates. Small substrates were primarily used during feeding and movement. Oblique substrates were also used during both behaviors, while vertical substrates were more common only during feeding. These findings provide valuable information for habitat preservation and management, as well as captive breeding or rehabilitation programs.

The preservation, maintenance, or restoration of continuous complex forest canopies with a diverse network of small branches is essential for ensuring that animals perform well within their habitat to survive and reproduce. The protection or reforestation of native tree species that rapidly develop a layered canopy with abundant thin branches is of primary importance. Additionally, long-term habitat monitoring should be implemented to assess changes in forest structure and pygmy slow loris behavior, using these data to inform adaptive management. The involvement of local communities through education, alternative livelihood development, participatory forest management, and animal observation will reduce habitat degradation and ensure local support for conservation initiatives. Ultimately, the effective conservation of *X. pygmaeus* requires an approach that integrates behavioral ecology with landscape management, forest structure restoration, and community engagement.

## Figures and Tables

**Table 1:** Definition and description of the recorded variables for captive *Xanthonycticebus pygmaeus*.

Substrate Type	
Branch	Network of branches of different diameters
Artificial	Wooden nest box, ledges, feeders
Ground	Enclosure floor
**Substrate size (relative to foot span [12])**	
Small	Substrate fully grasped by a completely flexed foot (<20 mm in diameter)
Medium	Substrate partly grasped by a semi-extended foot (20–50 mm in diameter)
Large	Substrate cannot be grasped by a fully extended foot (>50 mm in diameter)
**Substrate inclination**	
Horizontal	Angle between 0° and 22.5°
Oblique	Angle between 22.5° and 67.5°
Vertical	Angle between 67.5° and 90°
**Behavior**	
Move	Body displacement through its surroundings
Feed	Search, acquisition, and processing of food items
Rest/pause	Inactivity for short or long periods
Groom	Care of external body surface on the same (auto-grooming) or different animal (allo-grooming)
Social	Social interactions between two or more individuals (e.g., spatial proximity, play, cooperation, agonistic encounters, vocal communication, etc.)
**Locomotor modes**	
Quadrupedal walking	Symmetrical slow/moderate (walk) and fast (run) progression along single horizontal and moderately inclined substrates
Vertical Climbing	Symmetrical quadrupedal ascent or descent along vertical or steeply inclined substrates
Clambering	Irregular pronograde or semi-pronograde quadrupedal locomotion across multiple substrates
Bridging	Gap crossing mode involving reaching action across distantly located substrates with irregular limb movements
Suspensory locomotion	Supinograde symmetrical slow/moderate or irregular asymmetrical below branch locomotion employing all four limbs
**Postural modes**	
Standing	Pronograde tri/quadrupedal posture with flexed, semi-extended, or fully extended limbs
Sitting	Orthograde or leaning seated posture with strongly flexed hind limbs
Bipedal	Above-branch standing on two moderately flexed limbs assisted by forelimbs
Clinging	Orthograde grasping posture with extremely flexed limbs and the head upwards or downwards on vertical or steeply inclined substrates
Cantilevering	Grasping feet secure the lower part of the body to a steeply inclined substrate while the trunk and forelimbs are extended horizontally
Hanging	Suspensory posture below a substrate, with the body in a flexible supinograde or semi-pronograde position, using all combinations of fore- and hindlimbs

**Table 2:** Percentages of substrate size and inclination use during the different behavioral contexts in captive *Xanthonycticebus pygmaeus* (*N* = sample number).

	Move	Rest	Feed	Groom	Social
**Substrate Size**	**(%)**	**(%)**	**(%)**	**(%)**	**(%)**
Small	45.24	32.66	42.65	24.96	19.25
Medium	24.52	24.43	27.49	11.29	16.26
Large	30.24	42.91	29.85	63.75	80.79
**Substrate inclination**	**(%)**	**(%)**	**(%)**	**(%)**	**(%)**
Horizontal	28.52	40.87	27.49	66.29	67.49
Oblique	43.87	39.84	42.42	25.60	20.20
Vertical	27.61	19.28	30.09	8.11	12.31
*N*	*7351*	*7826*	*2818*	*4199*	*1357*

**Table 3:** Percentages of substrate size and inclination use during the different locomotor modes in captive *Xanthonycticebus pygmaeus* (*N =* sample number).

	Quadrupedalism	Vertical Climbing	Clambering	Suspensory	Bridging
**Substrate Size**	**(%)**	**(%)**	**(%)**	**(%)**	**(%)**
Small	24.51	23.22	72.43	33.33	44.12
Medium	22.55	36.81	22.94	50.62	32.35
Large	52.94	39.97	4.63	16.05	23.53
**Substrate inclination**	**(%)**	**(%)**	**(%)**	**(%)**	**(%)**
Horizontal	80.64	0.00	17.47	65.43	17.65
Oblique	19.36	4.26	58.10	34.57	52.94
Vertical	0.00	95.74	24.43	0.00	29.41
*N*	*7953*	*4150*	*9277*	*1507*	*664*

**Table 4:** Percentages of substrate size and inclination use during the different postural modes in captive *Xanthonycticebus pygmaeus* (*N* = sample number).

	Sitting	Standing	Bipedal	Clinging	Cantilevering	Hanging
**Substrate Size**	**(%)**	**(%)**	**(%)**	**(%)**	**(%)**	**(%)**
Small	22.91	21.31	25.37	32.95	67.87	47.46
Medium	35.24	13.55	59.70	28.72	21.42	33.02
Large	41.85	65.13	14.92	38.33	10.71	19.52
**Substrate inclination**	**(%)**	**(%)**	**(%)**	**(%)**	**(%)**	**(%)**
Horizontal	44.05	67.57	20.90	0.00	0.00	40.63
Oblique	55.95	31.71	79.10	7.72	28.57	43.50
Vertical	0.00	0.72	0.00	92.28	71.43	15.87
*N*	*2340*	*11,124*	*694*	*2665*	*292*	*6436*

## Data Availability

The raw data supporting the conclusions of this article will be made available by the authors on request.
